# Everybody's Page

**Published:** 1892-07-02

**Authors:** 


### Everybody's Page.

Nervous and consumptive patients who go to Colorado for a cure are wise to live in the open air as much as possible, but are warned against taking much exercise. Excess of zeal in the latter direction more often than not is said to account for the deaths of consumptive patients.

Many facts hitherto supposed to be uncontrovertible are now being disputed. Among them is the tradition which has held its ground for so long that negroes are comparatively free from malaria. Want of sufficient observation has no doubt led to the long life of this tradition, which modern evidence is tending to destroy. Numerous doctors who have had experience in malarious districts have noticed that members of the black race are not only subject to intermittent fever, but occasionally suffer from a severe type of remittent fever.

At a recent discussion held in the United States asthma was pronounced to be the result of a reflex action, the real seat of trouble being in the nose, whereas it seems to be located in the lungs or bronchial region. One physician recommended the removal of patients to the mountains or seashore, pronouncing Colorado to be the best place in the States for asthmatic patients. In making this recommendation he appears to have overlooked the varying conditions of the complaint. Seldom do two patients find relief in a similar atmosphere, and the most unlikely places suit some. We knew a sufferer who in his younger days had been accustomed to the bracing air of Cornwall, and when afflicted with asthma in later years found much relief in a thick London fog!

Fowl language, not that usually associated with Billingsgate and its purlieus, is being raised to quite an honourable position amongst the languages of the world. It has already been recorded on the phonograph, and the uses to which it may be put in the future are somewhat awful to contemplate. A crowing cock in the early morning is trying to the nerves of most sleepers, and now science has placed in the hands of our enemies another engine of torture, and bids fair to make life unbearable. So far, however, the instrument has been put to a less terrifying use. The method adopted is as follows: The phonograph is placed in a hen house full of inveterate cacklers, and when the unhappy instrument has been nearly driven out of its senses by being cackled at for half an hour it is removed to another coop where it is made to repeat the conversation. The result is said to be marvellous; we presume in the direction of increasing the laying and sitting powers of the hens, and we can well believe it.

Disinfection has been used recently for an unusual, if not novel, purpose in Russia. Owing to the need for food for the starving peasantry, committees were appointed for the purpose of organising a collection of scraps left after meals in the large cities. Many such scraps were collected from hospitals as well as from social gatherings. Thus arose the alternative of conveying contagious diseases to the peasants whom it was intended to benefit, or of destroying the food. In the face of the terrible scarcity in the country, the latter alternative was clearly to be avoided if possible, so the question was referred to the Bacteriological Institution of Odessa for solution, which came to the rescue with the suggestion that morsels of bread left over by persons infected with contagious diseases should be dried at a temperature of 250 deg. F., or be submitted to a current of steam of similar temperature for at least one hour, when they would be thoroughly disinfected. Food treated in such a manner might not prove very palatable to ordinary folk, though no doubt appreciated by starving mouths.

The temperance movement in Japan is being strongly supported by the Temperance Society of that country, the members of which have petitioned the House of Representatives to increase the taxation of alcoholic drinks, with a view to lessening their consumption. There is, however, no reason to suppose that legislation will meet with greater success in the direction of decreasing drunkenness in Japan than it has met with in America.

In one respect, at least, Europe has learnt her lesson only too well from America. The climax of advertising has, one would hope, been almost, if not quite, reached in the proposition to utilise the banks of the Suez Canal as a source of income derivable from advertisements placarded on high hoardings. It has been stated with all seriousness that a "large and enterprising" firm has already tendered for the leasing of the hoardings. Unfortunately, enterprise is not always well directed.

Popular superstitions die hard. A boy was recently bitten by a dog which flew at him while being stroked. The parent could not rest till the dog was destroyed, being under the impression that if the dog were destroyed there would be an end of all fear of hydrophobia setting in. Magisterial assurance that this idea was erroneous, coupled with advice to take out a summons against the owner of the dog, at length prevailed over the superstition, and will, we hope, have restored happiness and confidence to the breast of the aggrieved parent.

Cholera epidemics apparently can be of great use. Such an epidemic was reported to have broken out a short while ago amongst the sharks which abound in the Indian Ocean, and it is supposed that the sharks became infected by eating the bodies of the British sailors who had died in Bombay Harbour and who were buried at sea. Unfortunately, the cholera seems now to be turning its attention more especially to human beings. It is not improbable that the ravages made in Europe by the influenza will ere long be increased by this scourge.

The objects of the Selborne Society must recommend themselves to all classes, if not to all ages, of society. None but the most hopelessly truant schoolboy can object to the preservation from unnecessary destruction of such wild animals and plants as are harmless, beautiful, and rare, and to the promotion of the study of natural history. The work of the Society, which depends for its efficacy on the help of the public to a great extent, only needs to be widely known in order to gain the support it deserves. There is much need for one of its aims, the protection of objects of interest or natural beauty, from which the average Briton seems unable to keep his hands owing to an undesirable longing to hand down to posterity his usually commonplace name.

The public has had its growl and has been listened to by Mr. Horace Sedger in a manner which it ought to appreciate. That many people are debarred from attending some of the best London theatres by reason of the high prices charged for the best seats is a theory which has carried partial conviction of its correctness to Mr. Sedger, judging from the decision at which he has arrived with a desire to meet the popular wishes. Until further notice the "Mountebanks" will be performed every Wednesday afternoon at half-price. The verdict, we should think, is likely to be no hesitating one, and if the public does not take advantage of this opportunity, it must have the grace in the future to smother ts growls, and cease to emulate the proverbial farmer.

